# Distinguished Features of Adaptive Strategies of Halophytes and Glycophytes with Different Types of Photosynthesis in Response to Climatic Stressors

**DOI:** 10.3390/plants15071014

**Published:** 2026-03-26

**Authors:** Zulfira Rakhmankulova, Kristina Toderich, Kinya Akashi, Elena Shuyskaya

**Affiliations:** 1 K.A. Timiryazev Institute of Plant Physiology of Russian Academy of Science, 127276 Moscow, Russia; zulfirar@mail.ru (Z.R.); evshuya@gmail.com (E.S.); 2 International Platform for Drylands Research and Education, Tottori University, Tottori 680-0000, Japan; akashi.kinya@tottori-u.ac.jp; 3 Institute of Agrobiotechnologies and Food Security, Samarkand State University, Samarkand 140104, Uzbekistan; 4 Faculty of Agriculture, Tottori University, Tottori 680-8553, Japan

**Keywords:** C_3_ species, C_4_-NAD-ME and C_4_-NADP-ME species, elevated CO_2_, elevated temperature, drought, drylands

## Abstract

Extreme weather events such as higher temperatures, droughts, and soil salinization are projected to increase as atmospheric CO_2_ concentrations rise and climate change progresses. These factors have a negative impact on global food security, the water supply, and ecosystem productivity. The focus of this review is on modern concepts, comparative studies, and our data on the mechanisms of adaptation of halophytes and glycophytes with different types of photosynthetic metabolism (C_3_, C_4_) to the individual and combined effects of climatic factors. The analysis revealed that C_3_ and C_4_ species and C_4_-NAD-ME and C_4_-NADP-ME species differ in terms of stability and photosynthetic plasticity. Under drought conditions, both individually and in combination with other factors, C_4_ halophytes demonstrate the advantages of efficient photosynthesis and salt tolerance. Halophytes with C_4_-NADP-ME are characterized by uniquely high levels of plasticity and variability in photosynthetic metabolism. This is reflected in their ability to mitigate the negative effects of elevated temperatures and drought through the use of elevated CO_2_ (eCO_2_). The mitigating effect of eCO_2_ on photosynthesis at elevated temperatures was not detected in halophytes, regardless of photosynthesis type. Halophytes possess an augmented capacity for heat tolerance. Integrating fundamental scientific knowledge with urgent practical needs will enable us to predict changes in ecosystems and create new, sustainable agricultural systems.

## 1. Introduction

Increasing concentrations of CO_2_ in the atmosphere and climate change may lead to more frequent and intense extreme weather events, such as rising temperatures, heavy rainfall, droughts, and salinization [1,2]. All of these factors have a negative impact on plant growth, development, and productivity [2,3,4,5,6], and as a consequence, on global food security, the water supply, ecosystem productivity, and the global carbon cycle [7]. The effects of climate change are becoming increasingly unpredictable. Plants in their natural environment rarely encounter abiotic stresses in isolation. Instead, they are usually exposed to the combined impact of several climatic factors simultaneously. These combined stress factors cause more complex and unpredictable plant responses than individual factors do [2,7]. Furthermore, combined abiotic stresses often occur gradually and are sublethal, i.e., they are relatively weak compared to severe stresses [2]. The combination of weak actions and factors can induce significant acclimation responses, enabling plants to optimize their metabolism under suboptimal conditions [2,8,9]. In recent years, a large amount of scientific research has been conducted into the mechanisms by which plants adapt to complex environmental factors. It has been established that plant responses occur at various levels of organization, which are regulated by highly coordinated, complex molecular networks [2,10].

Plant growth and productivity are largely determined by photosynthesis, a fundamental and multi-stage physiological process [11,12,13]. Photosynthesis involves light reactions, also known as the electron transport chain (ETC), and dark reactions (Calvin–Benson–Bassham cycle). These reactions are controlled by numerous genes/gene products encoded by either chloroplasts or the nucleus. Gene expression in both cellular compartments is highly dynamic and dependent on environmental factors [14,15]. Abiotic factors such as high temperatures and water deficit affect plant growth, development, and productivity by causing numerous biochemical, structural and physiological changes. A decrease in transpiration caused by stomatal closure, inhibition of photosynthetic enzymes and ATP synthase activity leads to a reduction in the activity of photosynthesis. Metabolism of proteins and membrane stability are disrupted, while oxidative stress increases [16,17,18,19].

The emergence of C_4_ plants was one of the most successful evolutionary responses to climate change. Although C_4_ species account for just 3% of angiosperm species, they are responsible for around 25% of net primary productivity on Earth [20,21,22,23]. Compared to C_3_ species, C_4_ plants are characterized by a number of biochemical and morphological features [21,22]. They have an effective carbon concentrating mechanism (CCM), which leads to increased drought and heat tolerance in C_4_ species [20,21,24,25,26,27,28]. However, opinions differ regarding the limited capacity of C_4_ species to withstand multiple concurrent climatic stressors [29,30,31,32,33,34,35]. Thus, the response of C_4_ plants to complex changes in climatic factors and resistance mechanisms is extremely diverse [35,36,37], and the limits of their resistance are unclear, especially under the combined impact of climate stressors [26,31,33].

Global climate change, particularly warming and drought, leads to increased evaporation and consequently contributes to secondary soil salinization [38,39,40]. Soil salinization is currently becoming an increasingly global problem that is seriously affecting the productivity of important agricultural crops worldwide. Halophytes, or salt-tolerant plants, are able to survive and complete their life cycle in highly saline environments (200–500 mM NaCl). These species have great potential for phytoremediation of saline soils, as well as for improving plant tolerance to salinity [41]. The identification and characterization of salt tolerance-related genes that encode signaling components in halophytes has enabled the development of transgenic crops with improved salt tolerance [42]. However, the widespread use of wild halophytes as a potential model system for studying salt tolerance is limited by the lack of complete genomic information and insufficient systematic study of their regulatory molecular mechanisms of tolerance [39]. Concepts regarding the tolerance of C_3_ and C_4_ halophytes to climate change are contradictory, and their physiological, biochemical, and molecular-genetic mechanisms have not been sufficiently studied [19,39,43,44,45,46,47,48,49,50,51,52].

Consequently, salinity, combined with climatic factors, seriously limits the growth and productivity of new potential food and fodder crops. Understanding the relationship between salt tolerance and photosynthetic metabolism, and the adaptive strategies that enable halophytes to survive in saline conditions, is of great scientific and practical interest.

This review discusses and analyzes modern concepts and comparative studies, as well as our own data on the adaptation mechanisms of halophytes and glycophytes with different types of photosynthetic metabolism to the combined effects of climatic factors. Our aim was to test the following hypotheses: (1) Plants with different types (C_3_ and C_4_) and subtypes (C_4_-NAD-ME and C_4_-NADP-ME) of photosynthetic metabolism exhibit different levels of tolerance and photosynthetic plasticity in response to the combined effects of climatic factors. (2) Salt-tolerant species have high resistance to other abiotic factors. (3) The unique ecological capabilities of salt-tolerant species are associated with the type of photosynthetic metabolism and its plasticity.

## 2. Halophytes and Their Physiological Responses to Salt

### 2.1. Halophytes

Climate change could affect the ability of global agricultural systems to provide food and fuel for the world’s population, especially as salt-affected lands increase [19,53]. Fewer than 1% of plant species can tolerate soil salinity [39,54,55,56]. Halophytes, or salt-tolerant plants, are able to survive in saline environments subjected to osmotic and toxic ionic stresses. The significant diversity in the ecology, morphology, anatomy, and physiology of salt-tolerant plants has led to a variety of definitions and classifications of “halophytes” [54,57,58,59,60]. Halophytes are divided into facultative, which can grow in both non-saline and saline soils, and obligate (“true”), which require a certain concentration of salts in the soil for optimal growth depending on their sodium requirement for growth and development.

**Figure 1:**
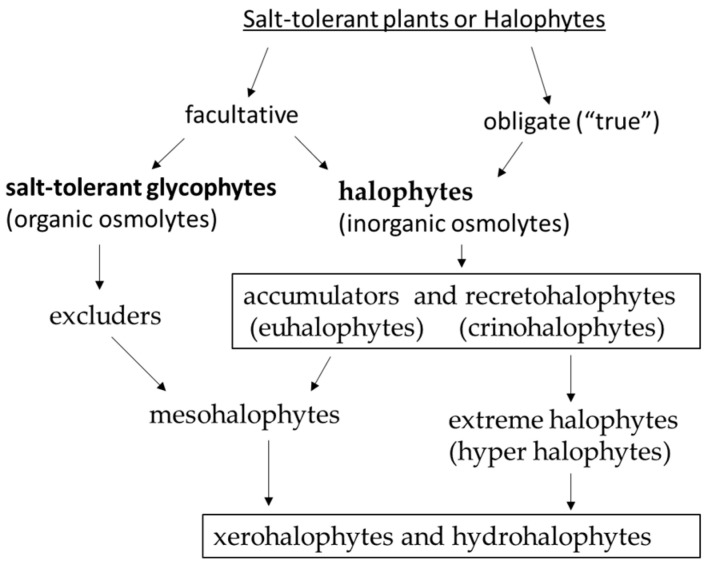
Schematic diagram of the classification of salt-tolerant plant species.

Salt-tolerant plants are divided into two groups based on the nature of their main osmolytes: salt-tolerant glycophytes (main osmolytes are organic osmolytes, such as low molecular weight compounds and amino acids) and halophytes (main osmolytes are inorganic ions Na^+^ and Cl^−^). The latter group includes salt accumulators (euhalophytes), which store and compartmentalize sodium and chlorine in the vacuoles of above-ground organs’ cells. This group also includes recretohalophytes (secretors, crinohalophytes), which secrete excess inorganic ions through salt glands and salt hairs onto the leaf surface. Halophytes can be categorized according to their habitats as hydrohalophytes (growing on wet or damp soils, such as sea coasts or wet salt marshes) and xerohalophytes (growing on dry soils in arid zones, such as deserts or semi-deserts) [39,54,56,57]. According to their degree of halophyticity, extreme halophytes (irreversible and reversible) and mesohalophytes can be distinguished [47,60] (Figure 1).

Significant differences were found between salt-tolerant monocotyledonous and dicotyledonous species. Most dicotyledonous halophytes grow optimally at 50–250 mM NaCl, while salt-tolerant monocotyledonous species generally prefer non-saline or slightly saline substrates (≤50 mM NaCl) [57].

The potential of halophytes as agricultural crops for saline soils, as well as for the bioremediation of degraded land (reclamation, phytoremediation, and phytodesalination), has recently been the subject of active investigation [55,56,61,62,63]. However, little data exists on the impact of salinity on their yield potential. The impact of salinity on fodder and grain quality varies depending on the species, although it often reduces quality [64]. Nevertheless, the overall impact of salinity on livestock production and livestock feeding has rarely been evaluated [55,64]. Furthermore, halophytes produce phenols at concentrations that give them high antioxidant and antimicrobial activity. This makes them ideal sources of bioactive molecules for a variety of industries [65]. Research has shown that repeated planting and harvesting of halophytes through phytodesalination can effectively restore saline land, converting it from wasteland into farmland [41,56,66,67]. A new concept, “circular halophytes mixed farming (CHMF),” is currently being developed. In this model, halophytes are cultivated alongside agricultural crops to manage the dynamics of soil, water, and plant salinity [39,64] (see Section 5).

### 2.2. Mechanisms of Salt Tolerance

Salinity causes two types of stress in plants: osmotic and ionic. It was previously thought that the effects of osmotic and ionic toxicity occurred at different times: general osmotic stress caused early responses, while sodium-specific responses were induced later [19,68,69,70]. However, the discovery of rapid salt signaling and the rapid sodium-induced response of root growth has challenged this concept [71,72]. The first plant responses to salinity have been found to occur within seconds to hours of salt stress [73,74]. Three majors early signaling compounds have been identified: glycosylinositol phosphorylceramide (GIPC, a sphingolipid), 3′,5′-cyclic guanosine monophosphate (cGMP), and reactive oxygen species (ROS) [72,73,74,75,76]. Osmotic adaptation involves altering ionic homeostasis by exclusion, accumulation, or excretion of ions through salt glands/bladders and the synthesis of osmoprotectants. Quaternary derivatives of amino acids, such as proline, glycine, glycine betaine, and α-alanine, as well as complex sugars such as raffinose, play an important role in osmotic regulation [19,61,77,78].

The decline in productivity under saline conditions is primarily caused by the negative impact of salinity on photosynthesis, which is typically associated with stomatal and non-stomatal limitations [79,80]. Salinity causes a decrease in leaf osmotic potential and stomatal closure, resulting in decreased stomatal conductance and photosynthetic rate. It also leads to the inactivation of photosystem II (PSII) reaction centers and the destruction of the oxygen-evolving complex. Furthermore, salinity decreases the electron transport rate and the maximum quantum yield of PSII [19,80]. Salinity also leads to an increased ROS level in plant tissues, resulting in oxidative damage to membrane lipids, proteins, and nucleic acids [19,81,82]. In order to neutralize high ROS levels, plants have developed an effective system of non-enzymatic and enzymatic antioxidants [19,39,46,61,83,84]. Salt-tolerant plants often experience lower levels of oxidative stress than salt-sensitive plants, which is associated with an effective antioxidant system [46,48,49,85,86].

Along with heat and drought, salinity is one of the main environmental conditions for the evolution of C_4_ plants [20]. C_4_ species have been shown to account for a particularly high proportion of the herbaceous flora of saline soils [87,88,89]. Salt tolerance is significantly more common among C_4_ grass species than in C_3_ grass species [90]. Within the Chenopodiaceae family, C_4_ photosynthesis likely evolved in salt-tolerant species [89,91]. The possible causal relationships between C_4_ photosynthesis and salt tolerance are investigated and discussed [90,91,92].

Salinity causes changes in the expression of many genes of various functional categories that are responsible for minimizing osmotic and ion-toxic effects [19,93]. These genes encode proteins that are associated with photosynthesis, the synthesis of osmolytes, membrane channels, and antioxidant enzymes, as well as signaling and regulatory elements, including transcription factors, such as bZIP, DREB, MYC, MYB, NAC, and WRKY, etc. Their significant correlation with salinity suggests that they have great potential to increase plant tolerance to salt stress [19]. Furthermore, halophytes have been shown to mediate salt tolerance by regulating stress-responsive genes through regulatory mechanisms, including abscisic acid [47].

In general, halophyte salt tolerance mechanisms include osmotic adaptation through altered ion homeostasis and synthesis of osmoprotectants, induction of antioxidants, and activation of genes involved in these pathways [19,39,47,94,95,96,97]. It is assumed that all plants have similar mechanisms for regulating salt tolerance, with quantitative rather than qualitative differences between halophytes and glycophytes. This may be due to the higher expression of key genes involved in the salt tolerance mechanism or higher activity of halophytic enzymes compared to corresponding glycophytic enzymes [47,96,97,98].

## 3. Types of Photosynthesis

### 3.1. C_3_ Photosynthesis

In most plants, photosynthesis occurs via the C_3_ pathway. In this process carbon dioxide (CO_2_) is fixed directly in the Calvin–Benson–Bassham cycle in mesophyll cells to form a three-carbon compound called 3-phosphoglycerate. Photosynthesis consists of the electron transport chain (ETC) and the Calvin–Benson–Bassham cycle. The ETC includes the pigment–protein complexes of photosystem II (PS II; EC 1.10.3.9) and photosystem I (PS I; EC 1.97.1.12) operating in two main modes. One of these modes is linear electron transfer (LET), which involves two photochemical reactions that carry out the photolysis of water and the formation of oxygen and the reduced form of Fd, NADPH, and ATP. The other mode is the cyclic electron transport around PSI (CET PSI), which involves only one photochemical reaction center and leads to the ATP formation [99]. Photosynthesis is a complex process integrated into changing conditions. The efficiency of the electron transport chain is closely coordinated with the activity of the Calvin–Benson–Bassham cycle enzymes. However, it is unclear how the intrinsic temperature sensitivity of the electron transport system will interact with that of carbon metabolism under changing climatic factors [99]. Analyzing this relationship is crucial for understanding how the C_3_ pathway functions and for the relative advantages and disadvantages of the C_3_ pathway compared to the C_4_ pathway. There are many quantitative models of C_3_ photosynthesis that facilitate the analysis of physiological parameters under stressful conditions. One promising model links the Cyt *b*_6_*f*-based description of the response to changes in CO_2_ concentrations [99,100].

### 3.2. C_4_ Photosynthesis

In C_4_ plants, the Hatch–Slack cycle involves the initial fixation of CO_2_ first in mesophyll cells as a four-carbon compound (oxaloacetate). This compound is then transported as organic acids to the bundle sheath cells, where it is released and utilized in the Calvin–Benson–Bassham cycle. This metabolic superstructure functions physiologically as a CCM (carbon concentrating mechanism), providing many of the benefits of C_4_ photosynthesis, such as higher photosynthetic efficiency and suppression of photorespiration, particularly under adverse conditions [20,21,24]. Plants with C_4_-type photosynthesis are divided into three large biochemical groups or subtypes depending on the decarboxylating enzyme: NAD-ME (aspartate), NADP-ME (malate), and PEPCK (PEP carboxykinase, aspartate) [20,21,22,24]. The NADP-ME subtype was the first C_4_ photosynthesis biochemical pathway to be studied. This pathway is used by important agricultural crops such as maize, sorghum, and sugarcane in the process of photosynthesis. Malate and pyruvate act as transport products [101]. In the NAD-ME subtype, the main transport product, aspartate, is synthesized in the cytosol of mesophyll cells, whereas malate formation and decarboxylation of NAD-ME occur in the mitochondria of bundle sheath cells [22,102,103]. PEPCK, as a type of C_4_ photosynthesis, is not found in pure form in plants. It functions as an auxiliary decarboxylase in plants with other subtypes of C_4_ photosynthesis [104]. Representatives of the various biochemical groups of C_4_ plants respond differently to stress. Plants with the NAD-ME subtype are considered to be more drought-tolerant [22,105,106] and salt-tolerant [92] than species with the NADP-ME subtype. For instance, a lineage of Chloridoideae grasses, predominantly composed of NAD-ME species, exhibits significantly greater increases in water use efficiency under drought conditions than grasses with the NADP-ME subtype [105,107]. Plants with the NADP-ME subtype tend to exhibit higher nitrogen use efficiency than other C_4_ species [22,108,109,110,111]. In addition, it has been shown that species with the NADP-ME photosynthetic subtype are more variable and plastic under stressful conditions than C_4_ NAD-ME species [92].

CET PSI is known to play a special role in the functioning of the ETC in C_4_ plants [112,113]. CET PSI consists of two distinct pathways: (1) a major PGR5/PGRL1 pathway (PGR5, which is dependent on proton gradient-5 and PGR5-like photosynthetic phenotype (1)) and (2) a minor pathway dependent on the chloroplast NADH dehydrogenase (NDH) complex [112,113,114]. It is proposed that the NDH-mediated CET pathway largely supplies the additional 2 ATP molecules required for C_4_ CCM function [112,115,116]. In C_4_ species, the activity of NDH-dependent CET activity is higher than PGR5/PGRL1 [115], and the content of the NDH complex can be 10-fold higher than in C_3_ species [112,116]. The key factor in determining the electron transport is ferredoxin, which exists in plants in two isoforms (FDI and FDII). FDI stimulates LET and is localized in mesophyll cells, whereas FDII activates CET PSI in bundle sheath cells and is required for C_4_ CCM [113,117]. Furthermore, it is suggested that CET PSI is involved in plant tolerance mechanisms by balancing the need for ATP and NADPH during adaptation of the photosynthetic apparatus to changing environmental conditions [118]. Adverse conditions (e.g., drought, cold, heat, and low/high light) have been shown to activate both the PGR5/PGR5L1 and NDH CET pathways, but the preferred pathway varies depending on the plant species [119,120,121].

Despite the increased interest in these plants in the context of food security under ongoing climate change, there is still no consensus regarding the drought and heat tolerance of C_4_ species [25,27,28,30,31,33,34].

The ability to tolerate salts has evolved independently in various families, including those with C_4_ photosynthesis. However, the observed link between the evolution of C_4_ photosynthesis and salt tolerance may simply be due to the peculiarities of the phylogenetic distribution of halophytes and C_4_ species. A phylogenetic analysis of the relationship between the photosynthetic pathway and salt tolerance in Poaceae grasses and salt tolerance revealed that salt tolerance is significantly more prevalent in C_4_ lines than in C_3_ lines [90]. In C_3_ and C_4_ halophytes of the Poaceae and Amaranthaceae families, C_4_ species were detected to exhibit stronger osmotic adaptation and greater salt tolerance than C_3_ species [122,123]. In the subfamily Chenopodiaceae (family Amaranthaceae), leaf/stem succulence is one of the adaptations to salinity, due to the development of water-storage cells around the vascular bundle. This adaptation is present in both C_3_ and C_4_ species. Moreover, Kranz cells are located around water-storing mesophyll cells, rather than the vascular bundle, indicating that C_4_ syndrome emerged after succulence [89,124,125]. Furthermore, it has been suggested that the acquisition of C_4_ photosynthesis by Chenopodiaceae species may represent an adaptation of halophilic flora photosynthesis to seasonal soil desalination and drying [91].

## 4. Mechanisms of Adaptation of C_3_ and C_4_ Plants Under Individual and Combined Action of Climatic Factors

Environmental factors can significantly limit plant growth and productivity. The physiological response of plants to abiotic stresses involves a complex series of processes. This begins with the perception of stress and triggers a cascade of molecular events that occur at the physiological, metabolic, and developmental levels [19,126]. During the adaptation process in plants, changes are observed in a whole complex of processes, including the light and dark reactions of photosynthesis, cell wall composition, nutrient translocation, gene transcriptional activity, metabolite and lipid profiles [127,128,129,130,131,132]. Protective and antioxidant resistance mechanisms are activated [133,134]. All changes caused by abiotic stresses lead to a systemic imbalance of metabolic and energy processes. Redirecting energy and nutrients to defense mechanisms results in a decrease in growth rate, and, consequently, in a decline in biomass and grain production [19].

### 4.1. Elevated Concentrations of Atmospheric CO_2_

In recent years, a great deal of information has been accumulated on the effects of elevated CO_2_ (eCO_2_) concentrations on plant growth and productivity. eCO_2_ has generally been shown to improve photosynthesis rates, plant growth, and yield [19,26,37,135,136,137,138]. It is believed that C_3_ species exhibit a greater positive effect of eCO_2_ compared to C_4_ crops [139]. Due to their efficient CO_2_ fixation and CCM in the sheath cells, C_4_ plants usually show less stimulation of photosynthesis and growth under eCO_2_ compared to C_3_ plants [33,37,140,141,142]. However, other studies have found no moderating effect of eCO_2_ on C_3_ glycophyte (barley) [34,35] and C_3_ halophyte (quinoa) under drought, associated with increased dark respiration and reduced antioxidant enzyme activity [52]. Conversely, C_4_ species have demonstrated that eCO_2_ effectively stimulates photosynthetic metabolism [143] through higher water use efficiency (WUE) [144]. Additionally, ultra-high CO_2_ concentrations have induced a decrease in the rate of photosynthesis in both C_3_ and C_4_ species [145,146]. There is currently no consensus on whether eCO_2_ mitigates the negative consequences of drought and elevated temperatures in C_3_ and C_4_ species with different salt tolerance levels.

### 4.2. Drought

A water deficit is a critical abiotic stress that affects plant growth and yield productivity [147,148]. Water typically constitutes 50 and 90% of a plant’s fresh mass, with most of it (60–90%) located inside the cells and the remainder mainly in the cell walls [19,149]. Plants experience water deficiency when the transpiration rate from the leaf surface exceeds the water absorption rate by the roots. This disrupts the normal plant functioning, particularly leading to a decrease in stomatal conductance and the accumulation of osmolytes, antioxidants, and other protective compounds. It also leads to a decrease in photosynthesis rate and growth [150,151,152,153]. Moderate water deficiency causes significant morphological and physiological changes, while severe deficiency can lead to plant death [154]. The duration of plant reactions depends on the length and severity of the water deficit, as well as on the species, age, and ontogenetic developmental stage of the plant [155]. Plants have developed various adaptation and acclimatization strategies at different levels to maintain water balance [156]. Drought tolerance depends on the ability of plants to support physiological activity under water deficiency through altered gene regulation and metabolic pathways that reduce stress-induced damage [19,157,158].

An important indicator characterizing the water balance of plants is water use efficiency (WUE), which is defined as the amount of carbon assimilated as biomass or grain yield per unit of water used [19,159,160]. Identification of genomic regions controlling WUE revealed quantitative trait loci (QTL) associated with carbon isotope discrimination, transpiration, stomatal conductance, leaf temperature, and so on [19]. In the C_3_ plant Arabidopsis, the genetic basis of WUE was revealed through the assessment of δ^13^C [161,162]. The *ERECTA* gene was identified as being responsible for variation in WUE, primarily due to its effects on stomatal density, the epidermis, and stomatal conductance [162]. Similarly, a single naturally occurring amino acid substitution in MITOGEN-ACTIVATED PROTEIN KINASE 12 significantly reduced WUE in Arabidopsis by reducing stomatal response [26]. C_3_ and C_4_ plants exhibit different responses to drought at the cellular level. Drought suppresses stomatal development in C_3_ species but has little effect on stomata in C_4_ plants. These differences may be related to divergent expression of their *SPEECHLESS* genes. Notably, C_4_ cultures have evolved multiple *SPEECHLESS* homologues with distinct genetic structures and expression levels [27].

There is currently no consensus regarding the drought tolerance of C_4_ plants. Due to CCM, C_4_ plants are thought to be able to minimize stomatal conductance, leading to greater drought tolerance than C_3_ species [27,37,163,164], although the limits of their tolerance remain unclear [33]. However, there is also evidence that C_4_ species have weaker drought tolerance than C_3_ species, despite having higher WUE values [30,34,35]. Drought has been shown to negatively affect the light and dark reactions of C_4_ photosynthesis [34,35,147]. Some authors suggest that selection pressures have driven C_4_ plant diversity. Such plant diversity is achieved through adaptive changes, primarily in the hydraulic system, which are aimed at increasing WUE rather than improving carbon fixation [165]. While some researchers consider drought tolerance in C_4_ plants to be a species-specific trait, this view is not universally accepted [30]. Consider, for instance, the decline in the apparent photosynthetic rate of maize. This decline has been linked to restrictions in stomatal CO_2_ diffusion, diminished CO_2_ saturation around Rubisco and PEPC, and reduced carbonic anhydrase (CA) activity [148,166,167].

Comparing some C_3_ and C_4_ halophytes revealed different drought tolerance strategies. The C_3_ halophyte *Karelinia caspica* accumulates water (it is succulent), whereas the C_4_ halophyte *Atriplex tatarica* reduces water loss (it has a higher WUE) to survive in dry and saline conditions [168]. Meanwhile, the C_4_ halophytes *Atriplex halimus*, *A. nummularia*, *A. portulacoides*, as well as C_3_ halophyte *A. prostrata*, displayed similar tolerance to water deficits on average [169]. Thus, the relationship between the C_4_ syndrome and salt-and drought tolerance remains unclear. Therefore, studying the adaptive strategies of C_4_ species, which are characterized by resistance and high productivity under stress—particularly C_4_ halophytes, which have mechanisms that tolerate osmotic and ionic stress—is a relevant and promising task.

### 4.3. Elevated Temperatures (eT)

Rising average annual temperatures pose a serious threat to plant growth and productivity [2,37,170,171]. Temperature increases under natural conditions often occur gradually and smoothly, only a few degrees above the ambient temperature range, yet even these temperature changes trigger plant responses [2,172]. The first responses are reflected in morphological changes, such as an increase in the root biomass and hyponasty (an increase in the angle of leaf inclination), as well as a decrease in leaf area thickness and the number of stomata. These morphological and structural features help plants to avoid water loss due to evapotranspiration [19,173,174]. The eT shortens the plant life cycle by decreasing the duration of different phenophases in C_3_ and C_4_ species [37,175,176]. Long-term exposure to high temperatures has been reported to enhance photorespiration in plants [25,37]. Plants respond to heat stress (eT) by activating several defense mechanisms, including synthesizing heat shock proteins (HSPs), accumulating antioxidants, changing membrane fluidity, and gene expression, particularly Rubisco activase [35,177,178]. However, long-term exposure to high temperatures can lead to growth and development inhibition and even irreversible damage to plant tissues [35].

The eT rapidly induces the expression of heat shock transcription factors (HSFs) such as NAC, MYB, WRKY, RAV, bZIP, AP2/ERF, and ZAT, which regulate the expression of heat stress-responsive genes. This includes the accumulation of heat shock proteins (HSPs), which are protective proteins including Hsp100, Hsp90, Hsp70, Hsp60, and some MAPKs (mitogen-activated protein kinases) [179,180,181]. Transcription is activated by the binding of TFs to the cis-elements (ARE, CORE, W-box, GCC box, and as-1-like, etc.) in the promoters of these stress-inducible genes [19,182]. Heat stress-responsive genes such as NADPH oxidase (Rboh), dehydration-responsive element-binding protein 2A (DREB2A), heat shock factors (HsfA2, HsfA7a, and HsfBs), multiprotein binding factor 1C (MBF1C), and MAPK are regulated by HSFs. This promotes the resumption of normal cellular and physiological activity while reducing cell damage [2,183]. In Arabidopsis, heat perception has been shown to be partly mediated by the phytochrome B (phyB) photoreceptor, which controls the expression of a subset of heat-responsive genes [2,184,185]. A mediator involved in thermomorphogenesis is PHYTOCHROME INTERACTING FACTOR 4 (PIF4), which acts as a primary transcription factor that regulates downstream responses [186,187,188]. Along with PIF4, PIF7 is also implicated as a critical regulator of thermomorphogenic responses and is considered a true thermoreceptor [2,189,190,191].

The eT effect on photosynthetic capacity, including the light and dark reactions of photosynthesis, is a complex process and depends on both the initial leaf temperature and the degree of warming [35,192,193]. Heat stress negatively affects cell membrane thermal stability, and also, like drought, it affects stomatal conductance, osmotic regulation, and photosynthetic enzyme activity [194,195,196]. The Rubisco typically operates efficiently in the temperature range of 20 to 30 °C. When these conditions are exceeded, the rate of photosynthesis typically decreases due to limited activity of the electron transport system and Rubisco [19,25,100]. eT affects various components of the ETC differently: LET usually decreases after the thermal optimum is reached [25]; CET PSI is stimulated relative to LET [100,197]; NPQ increases [198]; the plastoquinone pool becomes more oxidized [199]; and the Cyt *b*_6_*f* turnover constant increases [200].

C_4_ species have evolved metabolic strategies, such as CCM, which enable them to respond to unfavorable conditions and ensure greater efficiency of Rubisco and photosynthesis in general, even at extremely high temperatures, compared to C_3_ plants [19,25,28,201]. However, a decrease in PSII function and apparent photosynthesis was observed in C_4_ sorghum under eT [202]. Furthermore, high temperature acclimation in C_4_ species is largely associated with biochemical changes. In particular, C_4_ plants grown at eT exhibit lower Rubisco and carbonic anhydrase activity [203]. A decrease in Rubisco activity may be associated with reduced ribulose bisphosphate regeneration and Rubisco activase activity [145,176,204,205,206]. In C_4_ halophytes (*Kochia prostrata*), acclimation to eT can lead to an increase in the dark respiration intensity (Rd) and potassium ion content, as well as changing the role of sodium and potassium ions and proline in salt tolerance mechanisms [207]. The accumulation of anions and cations in response to high temperatures allows halophytes to adapt osmotically to increased transpired water [208].

A decrease in PSII efficiency at elevated temperatures may be accompanied by an increase in the expression of genes encoding PSII components and LET, as well as Rubisco [209]. In the halophyte *Halimione portulacoides* (C_3_), eT resulted in a decrease in the maximum rate of electron transport, an increase in the rate of RC closure, a decrease in the quinone pool, and a decrease in connectivity between PSII antennae, all while inhibiting electron transport. This was more pronounced in the donor region of PSII, as it is a consequence of damage to the oxygen-evolving complex [210]. The effect of damage to PSII due to decreased oxygen-evolving complex activity has been described in other studies [211].

Significant differences in the thermal stability of light and dark reactions of photosynthesis were revealed in C_3_ and C_4_ halophytes depending on their adaptability to salinity. For instance, salt-adapted C_3_ (*Artemisia anethifolia*) and C_4_ (*Atriplex centralasiatica*) plants maintained notably higher *F*_v_/*F*_m_ values and net CO_2_ assimilation rates than non-adapted plants at temperatures exceeding 42 °C. This increased thermotolerance is associated with improved thermotolerance of PSII reaction centers, oxygen-evolving complexes, and the light-harvesting complex [212,213].

Heat stress disrupts redox homeostasis, leading to the formation of ROS and causing oxidative stress. Increased ROS induces the oxidation of molecules, membrane destruction, enzyme inactivation, and changes in gene expression [182]. At eT, both enzymatic and non-enzymatic antioxidants are involved in the detoxification of excess ROS [214]. Oxidative stress can lead to epigenetic changes, such as modification of histones that regulate gene expression [19,183,215]. Genes encoding Rubisco activase and antioxidant enzymes involved in the ROS detoxification process are promising candidates for the development of heat-tolerant crops [19,215,216].

### 4.4. Combined Action of Abiotic Factors

During climate change, stresses caused by factors such as eCO_2_ and warming do not occur in isolation; these stresses act simultaneously [23,217,218]. Individual climatic factors often interact synergistically, antagonistically, or cumulatively (additively) [7,219,220]. Furthermore, plant responses can vary greatly; for instance, photosynthetic responses to eCO_2_ and eT can be synergistic in certain conditions but antagonistic in others [23,221,222,223,224].

The response to combined stress depends on the species, the plant developmental stage, the type of photosynthetic metabolism, and the characteristics of the acting factors [7,219,220]. For example, the response of plants to the individual action of eT or drought alone largely depends on the type of photosynthesis (C_3_ or C_4_). C_3_ species experience less yield loss to eT than C_4_ plants, while C_4_ species are more resistant to drought. However, the combined effects of eT and drought on yield do not differ significantly between C_3_ and C_4_ species [35].

When studying how plants adapt to combined stresses, it is important to consider the number, complexity, and dosage of the stresses to which the plants are exposed. Even slight effects from multiple stressors can still have rapid and severe consequences for plant growth, productivity, and ecosystem stability [2,7,225]. In general, the morphophysiological and molecular response to a combination of some stresses is mainly determined by the most severe stress factor [2,226]. The duration of exposure to stressors also plays a major role [2,227,228,229]. The order in which factors act may also be crucial in determining the size of the plant response. When plants encounter a combination of sequential stresses, even weak initial exposure can induce priming or memory effects, altering responses to future challenges, a process known as cross-acclimation [219,230,231,232]. It has also been established that the morphophysiological response to combined stress depends on the severity of each stressor. For instance, in Arabidopsis, sublethal high temperature (27 °C) combined with progressive drought results in stomatal closure via a “gas-and-brake” regulatory mechanism. High temperature activates TARGET OF TEMPERATURE 3 (TOT3) kinase, which promote stomatal opening via the H+-ATPase (ARABIDOPSIS H+-ATPase 1, AHA1). Under drought conditions, OPEN STOMATA 1 (OST1) phosphorylates TOT3, which inhibits stomatal opening [2,233]. Comparative transcriptional and metabolic analyses of plant responses to combined and individual stresses show that stress combinations trigger specific signal transduction pathways in plants that differ from those activated by individual stresses. In addition, there is cross-talk between pathways and interactions between different metabolic components, which complicates the study of adaptive mechanisms [7,136,227,234].

When studying plant TFs and their functions in adapting to the combined effects of stressors, one of the largest TF families, NAC (NAM, ATAF1/2, and CUC2), is often analyzed. This family is involved in regulating resistance in various species [2,235,236,237]. Transcriptome analysis of Arabidopsis revealed a significant increase in HSFs, with their regulation differing under combined and individual heat and drought stress. These differences were mainly related to the expression of the HsfC1 level and the presence of HsfA6a, HsfA2, and HsfA37 transcripts [238]. A meta-analysis identified 340 genes that were transcriptionally upregulated in common during combined drought and heat [238], salinity and heat [239], and high light and heat [240] treatments in Arabidopsis [241]. Among these transcripts, TFs belonging to the HSF, MYELOBLASTOSIS (MYB), and ETHYLENE RESPONSIVE FACTOR (ERF) families were significantly increased. Moreover, the distinct expression patterns of these TFs under combined stress as opposed to individual stresses suggest that the transcriptomic responses of plants to each stress combination may be regulated by unique, specialized TFs. This may occur through the additive, subtractive, or combinatorial effects of the expression (patterns) of different TF groups, creating a distinct overall TF signature that is unique to a combination of stresses [2,242].

Currently, there is no consensus on the mitigating effect of eCO_2_ on the negative impacts of drought and eT. A mitigating effect was demonstrated on C_3_ glycophytes under eT and water deficit conditions [243], whereas no such mitigating effect was observed in C_3_ halophytes [52]. C_4_ plants exhibit a more adaptive mechanism for regulating stomatal conductance under the combined action of eCO_2_ and drought or eT than C_3_ species due to the presence of CCM. This ensures low stomatal conductance and increases and/or stabilizes WUE [26,28]. The improved thermotolerance of C_4_ plants under eCO_2_ may be associated with increased respiratory metabolism and the activation of protein and metabolite biosynthesis [244,245]. In the C_4_-NADP halophyte *Kochia prostrata*, eCO_2_ mitigated the negative impact of eT and water deficit on CO_2_/H_2_O gas exchange (apparent photosynthesis, transpiration) but enhanced their negative impact on PSII functioning. It also contributed to a significant increase in proline content and the activation of antioxidant protection involving catalase, phenolic compounds, and CET PSI [155].

It has been found that eCO_2_ activates different salt tolerance mechanisms in halophytes with different types of photosynthesis [246]. eCO_2_ supported photosynthesis in both C_3_ (*Chenopodium quinoa*) and C_4_ (*Atriplex nummularia*) species; however, C_3_ species remained significantly less salt tolerant than C_4_ species. In C_3_ species, protection against oxidative stress was achieved by mitigating the limitation of photosynthesis by stomata, resulting in a decrease in ETR/A_gross_. In C_4_ species, eCO_2_ did not stimulate photosynthesis. The decrease in ROS formation in C_4_ species was associated with a decrease in electron transfer in the ETC; in other words, there was an indirect non-stomatal effect [246].

The complex interaction between eCO_2_ and other climatic factors causes metabolic changes in halophytes with different types of photosynthesis. Exposure to eCO_2_ in combination with drought and salinity and eT resulted in changes to the lipid profile of *Salicornia ramosissima* (C_3_) [247], while exposure to eCO_2_ in combination with drought and salinity resulted in changes in the metabolite profile and increased antioxidant activity in some Suaeda species (C_4_) [6].

At the same time, some studies suggest that the beneficial impact of eCO_2_ on the negative impact of climate factors is exaggerated [35]. In fact, there is evidence to accept that eCO_2_ can have a negative effect on C_4_ photosynthesis under heat and water stress [33,248]. The ambiguity of responses to the combination of eCO_2_ and elevated temperature or drought in C_4_ species is thought to be due to species specificity [249], the diverse effects of these factors on transpiration [250], or a decrease in temperature-sensitive photosynthesis parameters, such as apparent photosynthesis, stomatal conductance, and PSII efficiency [29,155].

## 5. Comparative Analysis of the Adaptive Responses of Glycophytes and Halophytes (With C_3_ and C_4_ Types of Photosynthesis) to Climatic Factors (On Model Species)

Unlike glycophytes, halophytes have genetic and physiological mechanisms that enable them to survive and complete their full development cycle under saline conditions. Mechanisms that allow halophytes to cope with osmotic stress and ionic toxicity are believed to enhance their ability to adapt to changing environmental conditions. Salt-tolerant plants can accumulate salts in vacuoles or synthesize compatible solutes/osmolytes to maintain water balance and protect cells from damage. They also have mechanisms to stabilize cellular structure, which is important for maintaining normal metabolism under extreme conditions [39,43,45,47,51,251]. Furthermore, halophyte plants often exhibit less oxidative stress than salt-sensitive ones due to a more efficient antioxidant system [19,46,48,49,65]. However, some halophytes demonstrate sensitivity to drought [44,50,52] and to elevated temperatures [33,212]. Therefore, the question of halophyte tolerance to abiotic stress remains unresolved.

In recent years, a large amount of experimental material has been accumulated, and many articles have been published on the study of the mechanisms of complex stability of photosynthetic processes and plant productivity in response to the individual and combined action of two or three climatic factors in glycophytes with C_3_ [19,243] and C_4_ types of photosynthesis [33,37], as well as in halophytes with C_3_ [33,209,247,252] and C_4_ types of photosynthesis [6,207,212,253,254]. Comparative studies have also been conducted on the combined effects of two factors on C_3_ and C_4_ species of glycophytes [28,29] and halophytes [36,143,252,254]. However, there are far fewer studies that consider the combined effects of three or more climatic factors (eCO_2_, eT, drought, and salinity) on both C_3_ and C_4_ species simultaneously [35,255]. These types of studies allow for a more accurate comparative analysis of the adaptive responses of plants to climatic factors.

We conducted a comparative analysis using data from studies investigating the combined effects of three climatic factors—elevated eCO_2_ (eCO_2_), elevated temperatures (eT), and drought (D)—on C_3_ and C_4_ halophytes under identical conditions [52,155,256], as well as C_3_ and C_4_ glycophytes [35,255], which had a similar set of physiological parameters.

### 5.1. Comparison of Adaptive Responses of C_3_ and C_4_ Halophytes to Climatic Factors

To compare the adaptive responses to elevated CO_2_ concentration, eT, and D, individually and in various combinations, model salt-tolerant species used for fodder and food with different types of photosynthesis were investigated: *Chenopodium quinoa* (C_3_), *Kochia prostrata* (C_4_-NADP-ME), and *Amaranthus retroflexus* (C_4_-NAD-ME) [36,52,155,256].

**Figure 2:**
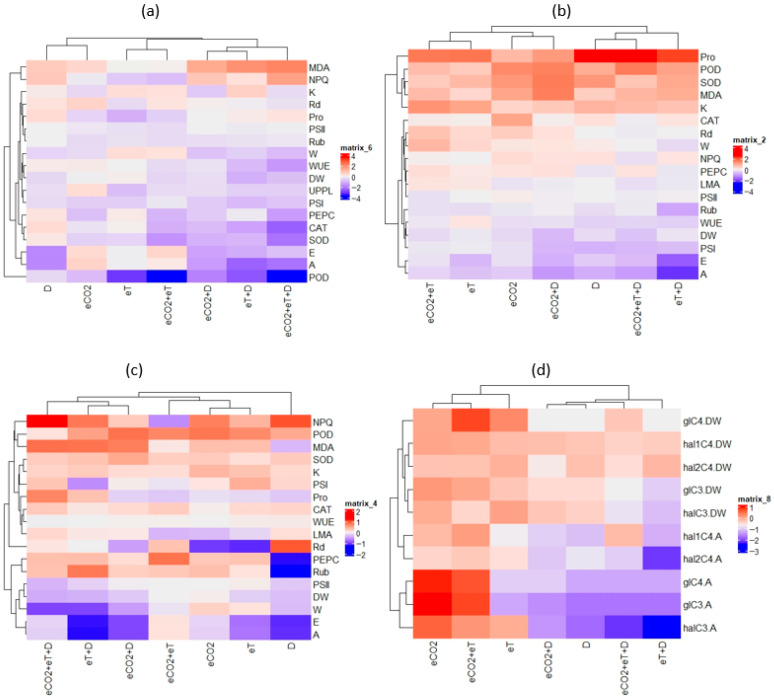
Heatmap changes in physiology-biochemical parameters in the following plants: (**a**) *Chenopodium quinoa* (C_3_), (**b**) *Amaranthus retroflexus* (C_4_-NAD-ME), (**c**) *Kochia prostrata* (C_4_-NADP-ME), and (**d**) in C_3_ and C_4_ glycophytes (gl) and halophytes (hal) [35,52,157,260] under individual and combined effects of drought (D), elevated temperature (eT), and elevated CO_2_ concentrations (eCO_2_) relative to control plants. Abbreviations: hal1C4—C_4_-NADP-ME; hal2C4—C_4_-NAD-ME; MDA—malondialdehyde content; SOD—superoxide dismutase, POD—peroxidase; CAT—catalase; Rub—ribulose-1,5-bisphosphate carboxylase/oxygenase (Rubisco) large subunit; PEPC—phosphoenolpyruvate carboxylase; PSI—activity of cyclic electron transport of PSI; NPQ—non-photochemical quenching; K—K^+^ content; A—apparent photosynthesis; E—transpiration intensity; WUE—water use efficiency; Rd—dark respiration; DW—dry biomass.

The selected C_3_ and C_4_ halophytes exhibited a similar sensitivity to the individual effects of D or eT on photosynthesis, a phenomenon that was more pronounced in the C_4_-NADP-ME species and was associated with limitations in stomatal and metabolic processes. However, changes in photosynthesis had little effect on plant growth in all model species, indicating their tolerance to D or eT (Figure 2). Similar results demonstrating stability in plant growth parameters under D were obtained for other C_3_ and C_4_-NAD-ME halophytes of the genus Atriplex [169]. At the same time, some studies have shown that C_4_ halophytes can be less resistant than C_3_ halophytes under water deficit conditions [168,257]. This may be due to the characteristics of the root system (C_4_-NADP halophyte *Atriplex tatarica*) and salt accumulation (e.g., C_3_ halophyte *Karelinia caspica*). The shallow root system of *A. tatarica* ensures lower transpiration water loss and stable leaf water potential, supporting this species to survive under water-deficit conditions. However, it reduces growth more than the C_3_ halophyte, whose strategy involves water accumulation [168].

Analysis of the combined action of two climatic factors (eT+D) on halophytes revealed a negative cumulative effect on the growth of C_3_ species and photosynthesis of C_4_-NAD-ME species (Figure 1). A similar behavior was described in the halophytes *Crithmum maritimum* (C_3_) [252] and *Amaranthus* species (C_4_ NAD-ME) [253].

No mitigating effect of eCO_2_ at eT was detected in C_3_ and C_4_-NAD-ME halophytes (Figure 2). However, it was observed in C_4_-NADP-ME species, which demonstrate high plasticity in photosynthetic and energy metabolism. The observed increases in photosynthesis and transpiration rates, reduced oxidative stress, and stable growth were likely due to increased PEPC content, enhanced adaptive dark mitochondrial respiration, and reduced dissipative non-photochemical costs in PSII (Figure 2). No mitigating effect of eCO_2_ on the negative impact of D was detected in C_3_ and C_4_ halophytes (Figure 2). Interestingly, an adaptive increase in the Rubisco and PEPC content did not result in positive changes to the photosynthesis and productivity of C_4_-NADP-ME species (Figure 2).

Climate change often involves three factors changing simultaneously: eCO_2_, eT, and D [6,247,258]. The mitigating effect of eCO_2_ on the combined action of eT+D for photosynthesis was only detected in C_4_ halophytes and was most pronounced in C_4_-NADP-ME species. However, elevated proline accumulation indicates increased osmotic stress in C_4_ species (Figure 2). A number of studies have also shown that the positive effect of eCO_2_ is weakened under the combined action of climatic factors [259,260,261].

**Figure 3:**
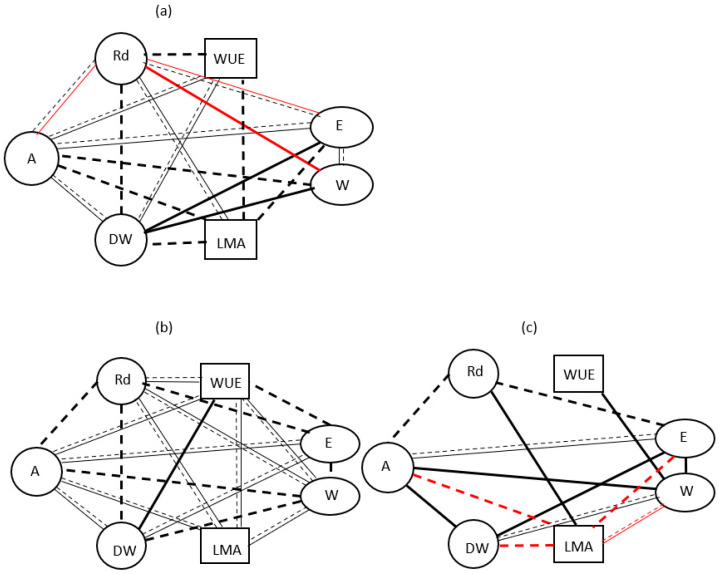
The degree of coordination between carbon and water metabolism based on correlation analysis in (**a**) *Chenopodium quinoa* (C_3_), (**b**) *Amaranthus retroflexus* (C_4_-NAD-ME), and (**c**) *Kochia prostrata* (C_4_-NADP-ME) in the adaptation to climatic factors (drought, elevated temperature, and elevated CO_2_ concentrations). Solid lines show the correlations at ambient CO_2_ concentration; dashed lines show the correlations at elevated CO_2_. Red lines indicate negative correlations. Thin lines correspond to r = 0.6–0.7, and thick lines correspond to r = 0.8–0.9. These diagrams are based on correlation analysis conducted using R software (v.3.6.1). A—apparent photosynthesis; E—transpiration intensity; WUE—water use efficiency; Rd—dark respiration; DW—dry biomass. W—water content; LMA—leaf mass per unit area.

A comparison of the consistency of changes in carbon and water exchange parameters (using correlation analysis) in model salt-tolerant species under the individual and combined action of eCO_2_, eT, and D showed that C_4_ halophytes differ from C_3_ halophytes in that they have a close relationship between water use efficiency (WUE, the ratio of apparent photosynthesis to transpiration) and leaf water content (W), as well as between W and leaf mass per unit area (LMA). In other words, WUE is more closely related to leaf water content and thickness than to photosynthesis and transpiration (Figure 3). The C_4_-NADP-ME species differed from the C_3_ and C_4_-NAD-ME species in that they exhibited greater variability in carbon and water exchange parameters, with few correlations observed between individual parameters (Figure 3).

At the same time, high water use efficiency did not result in high biomass productivity in C_4_-NADP-ME species, as evidenced by the lack of a relationship between biomass accumulation and WUE. Furthermore, only a relationship was found between biomass accumulation and CO_2_/H_2_O gas exchange indices (A and E) under ambient CO_2_ concentration (Figure 3). As no relationship was found between biomass accumulation and dark respiration intensity, it can be assumed that a significant increase in respiration associated with adaptation was observed alongside growth respiration (Figure 3). In contrast, high correlations were found between biomass productivity and WUE in C_4_-NAD-ME species and C_3_ species. However, this relationship of the C_3_ species was independent of CO_2_ concentration; in the C_4_-NAD-ME species, it only occurred at ambient CO_2_ concentration (Figure 3). In both species, most of the energy expenditure at eCO_2_ was likely associated with biomass accumulation, as indicated by the correlation between DW and Rd (Figure 3).

The analysis of adaptive reactions in response to climatic factors in C_3_ and C_4_ halophytes showed that different D options (D, eT+D, eCO_2_+eT+D) negatively affected the intensity of photosynthesis and the growth in all plants, regardless of photosynthesis type. At the same time, it was shown that C_4_ species had some advantages under unfavorable conditions, demonstrating greater stability of the photosynthetic apparatus than C_3_ species. This was probably facilitated by a more effective antioxidant system in C_4_ halophytes (Figure 1) [19,262] and a higher photosynthetic plasticity, most pronounced in the C_4_-NADP-ME halophyte, compared to C_3_ species (Figure 3), as confirmed by other studies [93,263].

### 5.2. Comparison of Adaptive Responses of Halophytes and Glycophytes with Different Types of Photosynthesis to Climatic Factors

To compare the adaptive responses of halophytes and glycophytes with different types of photosynthesis to eCO_2_, eT and D, individually and various combinations, we used the intensity of photosynthesis (CO_2_ assimilation, A) and productivity (dry biomass accumulation, DW) as the main physiological parameters. These parameters are often interrelated and largely determine the adaptive potential and final productivity of plants [11,261]. The table presents the results of the analysis, which allowed us to identify the characteristic features of the C_4_ plant adaptation (glycophytes and halophytes) and confirm existing knowledge. The first part of the table contains the general characteristics of halophytes and glycophytes with C_3_ and C_4_ types of photosynthesis, as previously described by many authors. So, C_4_ species are known to have high photosynthetic efficiency due to the CCM [20,21,24,25,26,27,28], antioxidant activity, which limits oxidative damage under stress [35,36,262,264,265]. The most plastic C_4_ species (halophytes and glycophytes) are those with the C_4_-NADP-ME type of photosynthesis [35,36,93,263].

Despite their high-water use efficiency, there is currently no consensus regarding the drought tolerance of C_4_ plants [30,34,35,37,163,164]. The table presents the results of our analysis of literature data and experiments on the reaction of glycophytes and halophytes with different types of photosynthesis to the action of climatic factors. Our study of the plant response to D showed that C_3_ and C_4_ glycophytes were less drought-tolerant than C_3_ and C_4_ halophytes, as evidenced by stable growth of the latter (Table 1) [34,35,52,155,256]. The eT reduces the intensity of photosynthesis in both glycophytes and halophytes with different types of photosynthesis. However, eT does not affect their growth (see Table 1) [25,35,52,155,256]. The low thermal stability of some C_4_ species may be associated with increased respiration (Rd), i.e., dissipation processes [29,32,35]. Furthermore, C_4_ plants have a higher temperature optimum for photosynthesis than C_3_ plants; their range is relatively narrow [25].

Physiological responses to combined stresses do not necessarily coincide with tolerance to individual stresses. This is because combined stress can induce unique and significant biochemical and molecular alterations, which often depend on adaptation to a specific combination of stresses and genotype [266]. The combined effects of eT and D (eT+D) also induce a complex plant response due to the molecular regulatory mechanism of “gas and brake” that controls the opening/closing of stomata in response to the simultaneous action of these factors [233,267,268,269]. According to some reports, the response to the combined effect of eT+D in C_3_ and C_4_ plants does not differ significantly [35,270]. Compared to the individual effect of these factors, the combined stress exhibits an additive/cumulative (negative) effect, increasing disruption to photosynthesis in both C_3_ and C_4_ species [9,253]. However, a comparative analysis of the combined effect of eT+D on glycophytes and halophytes with different types of photosynthesis showed that C_3_ species were less resistant, regardless of salt tolerance, exhibiting decreased plant productivity. The photosynthetic apparatus was sensitive only in C_4_-NAD-ME halophytes (see Table 1) [35,52,155].

**Table 1:** A comparison of the reactions of glycophytes and halophytes with different types of photosynthesis in terms of the intensity of CO_2_ assimilation (A) and plant productivity in relation to climatic factors.

Parameters and Protective Mechanisms	Glycophytes		Halophytes	
	C_3_ (Barley, Arabidopsis, etc.)	C_4_-NADP-ME (Sorghum, Corn, etc) C_4_-NAD-ME (Kleingrass)	C_3_ (Quinoa, White Goosefoot)	C_4_-NADP-ME (Kochia) C_4_-NAD-ME (Amaranth)
**Characteristics of C_3_ and C_4_ species**				
Efficiency of photosynthesis (under optimal conditions)	Less	More [104,271,272]	Less	More [36]
Antioxidant activity	Less	Higher [35,264,265]	Less	Higher [56,262]
Plasticity (variability) of morpho-physiological parameters	Less	Sorghum has more variability in its response to climate change than barley [35]	Less	C_4_-NADP-ME species are more variable in response to climate change than quinoa and amaranth [155]
**Climate factors**				
Drought (D)	Growth * and A are decreasing [35]	Growth and A are decreasing [34,35]	A is decreasing in quinoa, but growth does not change [52]	A is decreasing, but growth does not change [155]
Elevated temperature (eT+D)	A is decreasing, but growth does not change [35]	A is decreasing, but the growth rate remains unchanged [35] The temperature optima for photosynthesis in C_4_ plants are higher than in C_3_ plants, but their range is relatively narrow [25]	A is decreasing, but the growth rate remains unchanged [36,52]	A is decreasing, but the growth rate remains unchanged [36,155]
Negative cumulative effect of the combined action of eT+D	There is an effect on growth, but not on A [35]	There is no effect on either growth or A [35]	There is an effect on growth, but not on A in quinoa [52]	There is an effect on A, but not on the growth in C_4_-NAD-ME species There is no effect on either growth or A in C_4_-NADP-ME species [155,256]
**Mitigating effects of elevated CO_2_ concentrations**				
The mitigating effect of eCO_2_ on the negative effect of eT	eCO_2_ mitigates the effect of eT on A, but not on growth [35,139]	eCO_2_ mitigates the effect of eT on both A and growth [35,37]	The eCO_2_ has no mitigating effect [29]	The eCO_2_ slightly mitigates the effect of eT on A, but not on the growth of C_4_-NAD-ME species. The eCO_2_ mitigates the effect of eT on both A and growth in C_4_-NADP-ME species [36,155]
The mitigating effect of eCO_2_ on the negative effect of D	eCO_2_ mitigates the effect of drought on A, but not on growth [35]	In C_4_-NADP-ME species, eCO_2_ mitigates the effect of drought on A, but not on growth [35] In C_4_-NAD-ME species, eCO_2_ mitigates the effect of drought on both A and growth [273]	Not detected [52]	Not detected [155]
The mitigating effect of eCO_2_ on the negative effect of eT+D	eCO_2_ mitigates the effect of eT+D on A, but not on growth [35,256,274]	The effect of eT+D on growth is mitigated eCO_2_ in C_4_-NADP-ME species [35]	Not detected [52]	eCO_2_ mitigates the effect of eT+D on A, but not on growth [155]

* Growth was assessed by dry biomass; A—apparent photosynthesis (CO_2_ assimilation); eT—elevated temperature; D—drought; eCO_2_—elevated CO_2_ concentration.

The mitigating effect of eCO_2_ on the negative impacts of warming or D remains unresolved. In particular, stresses caused by eCO_2_ and warming often occur simultaneously under climate change [217,218], and photosynthetic responses to these stresses can be either synergistic or antagonistic depending on circumstances [23,36,221,222,244,245,249,250]. The ambiguity of plant responses to eCO_2_+eT is due to the fact that eT reduces the solubility of CO_2_ in water, which eCO_2_ can compensate for this factor [275,276]. Furthermore, eCO_2_ and eT have conflicting effects on stomatal limitation: eCO_2_ decreases stomatal conductance, whereas eT can enhance it [23,247,277,278,279,280]. The mitigating effect of eCO_2_ under eT is shown to be more pronounced in glycophytes and C_4_-NADP-ME halophytes (Table 1) [244,245]. The mitigating effect of eCO_2_ on the negative impact of D on photosynthesis was found only for glycophytes, regardless of the type of photosynthesis (Table 1) [35,52,155,256,273].

The question of the mitigating effect of eCO_2_ on the negative consequences of the combined action of D and eT (eT+D) in plants with different types of photosynthesis also remains unanswered. While the mitigating effect has been demonstrated on both C_3_ [243] and C_4_ [26,28,36] plants, there is information that an increased eCO_2_ concentration negatively affects C_4_ photosynthesis under heat and D, without affecting biomass [33,253]. It has been shown that the mitigating effect of eCO_2_ on the negative impacts of D and heat stress is stronger in terms of the physiology and yield of C_3_ crops than in C_4_ species. Notably, no positive effect of eCO_2_ was observed under eT+D in C_3_ halophytes, as evidenced by unchanged photosynthesis and productivity (see Table 1) [35,52,155,255,256,274].

To generalize the available information, a comparative analysis of the physiological and biochemical reactions of model fodder and food glycophytes (C_3_ barley and C_4_-NADP-ME sorghum) and halophytes (C_3_ quinoa, C_4_-NADP-ME *Kochia*, and C_4_-NAD-ME amaranth) to climatic factors was carried out using heat maps (Figure 2d). Glycophytes and halophytes were clearly divided into groups based on their physiological responses to climatic factors, depending on their photosynthetic metabolism. C_3_ species were less tolerant to the individual and combined effects of these factors than C_4_ species.

**Figure 4:**
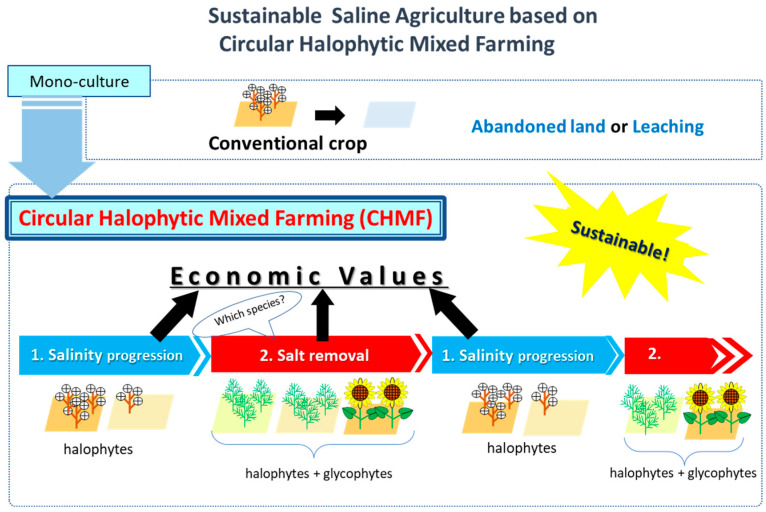
Bioremediation of salt-affected farmlands through cultivation of halophytes. High saline abandoned farmland (1) planted by halophytes with salt removal capacity alternated with salt tolerant glycophytes (2). After 3.5 to 8.0 years of cultivating various combinations of C_3_ and C_4_ halophytes and glycophytes, saline land becomes productive and suitable for growing salt-sensitive traditional crops.

The climatic factors were grouped into two categories based on the analysis of plant responses to them. The first group comprised different D variants (D, eT+D, and eCO_2_+eT+D), and the second group included the individual and combined actions of eCO_2_ and eT. The factors of the 1st group had the most negative impact on photosynthesis and productivity of all species; however, the C_3_ species were more sensitive. Moreover, C_3_ halophytes were less tolerant than C_3_ glycophytes to the combined action of eT+D and eCO_2_+eT+D. The exception was the C_4_-NAD-ME halophytes, which demonstrated sensitivity of photosynthesis to eT+D without affecting productivity. In general, halophytes showed greater stability in plant growth parameters under different D conditions. Overall, therefore, C_4_ species were more resilient to the action of D and eT factors of this group (Figure 4). Glycophytes were more sensitive to the climatic factors of the 2nd group, regardless of photosynthetic metabolism type. Photosynthesis was suppressed at eT, and it is stimulated at eCO_2_. Therefore, plant thermotolerance depended more on the salt tolerance than on the photosynthesis type (Figure 2d). The mitigating effect of eCO_2_ on photosynthesis and productivity at eT was more pronounced in glycophytes and was independent of photosynthesis type.

A comparative analysis of the combined effect of eT+D showed that C_3_ species were less resistant, regardless of salt tolerance, demonstrating a decrease in photosynthesis and productivity. The photosynthetic apparatus was also found to be sensitive in C_4_-NAD-ME halophytes. C_4_ species exhibited advantages under the combined influence of three factors (eCO_2_, D, and eT). The moderating effect of eCO_2_ under eT+D condition on photosynthesis was more pronounced in the C_4_-NADP-ME halophytes and on productivity in the C_4_-NADP-ME glycophytes. The advantages of C_4_ halophytes under various D conditions and associated factors are related to the combination of halophyte tolerance mechanisms and C_4_ photosynthesis characteristics. For example, the ability to resist osmotic and ionic stress enables them to more easily overcome osmotic shock. The presence of C_4_ CCM ensures relative independence from stomatal limitation of CO_2_ assimilation during photosynthesis and regulates plant water exchange. Furthermore, C_4_ species are characterized by a higher level of antioxidant protection (Figure 2d).

## 6. Prospects for the Domestication and Practical Use of Halophytes

Recently, halophytes have been widely utilized as agricultural crops in saline soils and for the bioremediation of degraded lands aimed to improve their productivity [55,56,62,281]. However, the data on selecting appropriate halophyte species for the phytoremediation of salinized soil is limited in the literature [282]. Halophytes with a higher degree of salt tolerance have better growth dynamics and greater structural plasticity, and seem to be more effective in phytoremediation. The ability to remove salts and the capacity of species to be phytoextracted are not the only factors affecting the uptake of salts and heavy metal ions from the soils. Several other issues must also be considered, including the chemical composition and concentration of salt, redox potential, pH, and organic matter content, among others [283,284]. The cultivation of C_3_ and C_4_ species, for example *Amaranthus retroflexus* (C_4_), *Atriplex nitens* (C_3_), *Kochia scoparia* (C_4_), and *Karelina caspia* (C_3_) in pure stands and in mixed trials on highly saline land yielded the biggest biomass production at the end of the vegetation period, while maximum salt uptake was detected during their active growth stage in summer [285]. *A. nitens* was the most promising species, removing salts from the soil at a rate of about 1.8 kg NaCl equivalent/kg-dry soil during the peak growth and beginning of flowering ontogenetic stages [282].

A wide range of genetic diversity in the halophyte species is being exploited to breed and select improved lines with enhanced salt tolerance, biomass production, and agronomic performance [55,286,287,288]. For example, quantitative trait loci mapping for nine agronomic traits has been reported in *Amaranthus hypochondriacus* [289]. A genetic study using F_1_ and F_2_ populations of *Amaranthus cruentus* and *Amaranthus* spp. revealed a single genetic locus for seed shattering on chromosome 2B [290]. Significant genetic diversity has been documented among natural populations in Atriplex, providing valuable genetic resources for breeding and selection [291,292,293].

Circular halophytic mixed farming (CHMF) increases the productivity of saline land. Long-term and year-round cultivation of C_3_ and C_4_ halophytes in CHMF returns promising results in terms of green biomass and seed production (Figure 4). This approach implies the continuous growth and subsequent disposal of halophytes’ aboveground plant tissues to reverse salinization levels and eventually reclaim desalinized land for alternative agricultural use. Succulent halophytes in particular accumulate higher levels of Na^+^ and Cl^−^ (3000–5000 mmol/kg) than other salt-tolerant species [251,294]. Results suggested the consecutive cultivation of the halophytes for 3–6 years would rehabilitate the high-saline farmland, allowing the growth of mung beans, sunflowers, foxtails, pearl millet, sorghum, and other glycophytes that are less salt-tolerant [295]. The aboveground biomass of salt accumulator halophytes was thought to be utilized as an alternative fuel, biocompost, and other useful products during the phytoremediation of salinized farmlands [296]. Previous studies emphasized the importance of incorporating neglected and underutilized crops (NUCs), including both C_3_ and C_4_ species, into saline dryland farming systems [297]. These species have been marginalized historically by intensive conventional agriculture. Promoting NUCs has become a key interest for farmers and agropastoralists, driven by their climate resilience and economic benefits, especially as livestock feed and grains for human consumption [281,298]. To maximize crop production after the phytoremediation, the continuous cultivation of halophytes reverses salinization levels and reclaims marginal lands for agricultural and other potentially economically beneficial uses. 

An equally important and promising area of application of wild halophytes is their potential use as carriers of genes for resistance to osmotic and ionic stress in the development of climate-resilient crops. C_4_ halophytes are particularly promising for this purpose, as they demonstrate a combination of salt tolerance and efficient photosynthesis under changing environments. Creating new crop varieties requires an interdisciplinary approach that integrates advanced technologies, such as multi-omics (transcriptomics, proteomics, and metabolomics) and functional genomics. This involves understanding the role of genes in the plant genome and their influence on plant functional traits and phenotype [2,299,300,301]. Accelerated selection, speed breeding methods, and synthetic biology, in combination with genome editing methods (CRISPR/Cas9), show promise and benefit [2,7,302]. Combining systems biology and artificial intelligence methods in combination with meta-analysis will provide insight into plant stress response mechanisms and interpret complex interactions between multiple stresses. This will enable the development of effective climate change adaptation and mitigation strategies [2,7,229]. To develop new plant varieties that are more resilient to climate change, it is necessary to identify the key breeding targets that balance the plant’s responses to multiple stresses while promoting growth recovery [225,280,303,304]. Additionally, advancing studies aim to identify TFs that play a pivotal role in how plants respond to combinations of stresses and to further explore photosystem II (PSII) [240] and PSI and its role in CCM in C_4_ species [305]. The mechanisms that maintain potassium homeostasis [306], the formation of stress-resistant metabolites under stress, and their potential use as markers in plant breeding [7,285,307] are also being studied.

## 7. Conclusions and Prospects for Future Research

Rising eCO_2_ and climate change are leading to unpredictable combinations of abiotic stress factors (D, eT, and salinity), which seriously affect plant growth and productivity. The combination of these stress factors dramatically reduces the efficiency of photosynthesis and crop yields, highlighting the need to develop resistant crop varieties. However, there is currently no consensus regarding the D and heat tolerance of C_4_ plants, despite their high water use efficiency. There is also very varied and often contradictory information on the mitigating effect of eCO_2_ on the negative impact of D and eT on C_3_ and C_4_ species. Recently, the potential of halophytes as agricultural crops for saline soils, for reclamation of degraded land, and as carriers of genes for resistance to osmotic and ionic stress has been actively studied. Meanwhile, it is assumed that halophytes and glycophytes have virtually identical mechanisms for regulating salt tolerance, differing only in the higher expression of key genes and the activity of salt-tolerance-associated enzymes in halophytes. Consequently, further systematic study of the molecular regulatory mechanisms of tolerance in halophytes is promising, despite the limited genomic information available [39].

**Figure 5:**
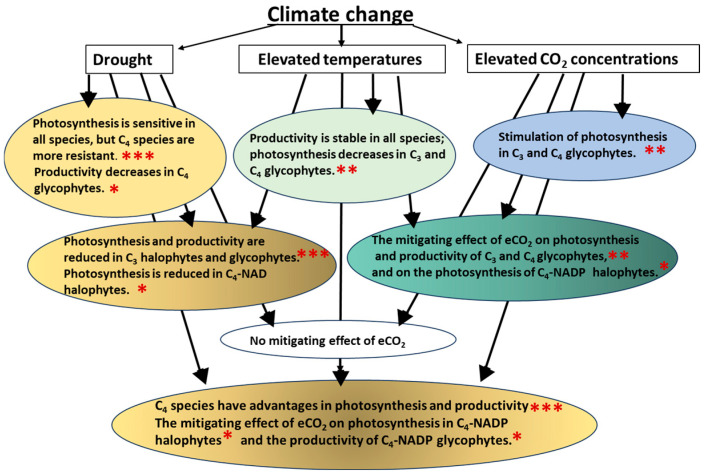
The impact of climate change on glycophytes and halophytes with different types of photosynthesis. *—Individual adaptive responses; **—Adaptive responses associated with salt tolerance; ***—Adaptive responses associated with the type of photosynthetic metabolism. Yellow: (1) different drought variants (D, eT+D, eCO_2_+D+eT) (1st group of factors). Green-blue: (2) eCO_2_ and eT (2nd group of factors).

An analysis of the results of various comparative studies, including our own, has enabled us to identify the characteristic features and universal adaptive strategies of halophytes and glycophytes with different types of photosynthesis in response to the impact of climatic factors (Figure 5). The analysis revealed that plants with different types (C_3_ and C_4_) and subtypes of photosynthetic metabolism (C_4_-NAD-ME and C_4_-NADP-ME) exhibit different levels of tolerance and photosynthetic plasticity in response to individual and combined climatic factors. Specifically, C_4_ halophytes and glycophytes had an advantage in D conditions, while both C_3_ and C_4_ halophytes performed well in condition eT. This phenomenon indicates that salt tolerance mechanisms play a more significant role in plant heat tolerance than the photosynthetic type.

Elevated CO_2_ levels are thought to have a more positive impact on C_3_ species. However, we found that the mitigating effect of eCO_2_ on photosynthesis was more pronounced at eT in glycophytes with different types of photosynthesis (C_3_ and C_4_), as well as in the C_4_-NADP-ME halophytes. The mitigation effect of eCO_2_ under the negative impact of D was not strong for all model species. Meanwhile, C_4_ species demonstrated benefits from the combined action of three factors (eCO_2_+eT+D). The mitigating effect of eCO_2_ at eT and D was observed in C_4_-NADP-ME halophytes on photosynthesis and in C_4_-NADP-ME glycophytes on growth.

The hypothesis that salt-tolerant species have a high potential for resistance to other abiotic external factors was confirmed only for the action of eT. Halophytes also showed a slight advantage under D conditions, but only those with C_4_ photosynthesis. Unlike C_3_ and C_4_-NAD-ME, a unique feature of C_4_-NADP-ME halophytes is their high metabolic plasticity and variability of photosynthesis, which is reflected in the mitigating effect of eCO_2_ on the negative effects of eT and D.

Thus, our analysis revealed that C_4_ halophytes are the most promising group of plants in a changing climate. Their unique ecological capabilities are closely linked to their type of photosynthetic metabolism and plasticity. The advantages of C_4_ halophytes under different D conditions, including the simultaneous action of three factors, are associated with a combination of halophyte tolerance mechanisms and the characteristics of C_4_ photosynthesis. These findings, obtained in model plants, require further, more detailed studies across a larger number of species. However, the insights described in this article enable us to formulate unanswered questions and identify prospects for further research. The following questions remain, in particular: How do plants coordinate physiological, biochemical, and molecular responses when subjected to multiple stresses? What are their tolerance thresholds for different combinations of stress factors? How do plants cope with oxidative stress under the influence of multiple factors? Similar questions have been raised by the authors of other studies [302]. Further studies aimed at identifying “general responses” independent of the stress type and “specific responses” associated with stress combinations, with significant overlap of TFs and signaling pathways, are promising [7,230,308]. Given that plants will predominantly respond to the primary stressor, including complex signaling pathways, it is necessary to understand the priority and dominance of responses to stress combinations [230,309,310], which can lead to stress memory and increased stress resistance [7,10,281,311].

## Data Availability

Data are contained within the article.
